# Spontaneous Left Anterior Descending Coronary Artery Dissection Requiring Coronary Artery Bypass Surgery

**DOI:** 10.21470/1678-9741-2017-0140

**Published:** 2017

**Authors:** Ana Paula Tagliari, Adriano Nunes Kochi, Luis Eduardo Paim Rohde, Orlando Carlos Belmonte Wender

**Affiliations:** 1 Department of Cardiovascular Surgery, Hospital de Clínicas de Porto Alegre, Universidade Federal do Rio Grande do Sul (HCPA-UFRGS), Porto Alegre, RS, Brazil.; 2 Department of Cardiology, Hospital de Clínicas de Porto Alegre, Universidade Federal do Rio Grande do Sul (HCPA - UFRGS), Porto Alegre, RS, Brazil.

**Keywords:** Coronary Artery Dissection, Spontaneous, Acute Coronary Syndrome, Coronary Artery Bypass

## Abstract

**Introduction:**

Spontaneous coronary artery dissection is a sudden separation between the layers of a coronary artery wall, non-iatrogenic or trauma related, that has been recognized as an important cause of myocardial infarction.

**Objective:**

To report an emblematic case, in terms of angiographic images, clinical presentation and predisposing factors, whose clinical management failure led to surgical intervention.

**Methods:**

A previously healthy 48-year-old male farmer was admitted to the emergency room complaining of anterior chest pain described as "tearing", which started after physical exertion. Anterior wall ST-segment depression was observed in the electrocardiogram and troponin levels were increased. The patient then underwent coronary catheterization. Angiography showed a tortuous left anterior descending coronary artery with a dissection line involving proximal and middle segments, resulting in mild to moderate luminal stenosis. At first, a conservative approach was chosen. Control cardiac catheterization, 3 months later, showed dissection progression to the distal segment.

**Results:**

The patient was referred to surgical treatment. Internal thoracic artery and a great saphenous vein graft were used to revascularize the target vessels. He had an uneventful postoperative course.

**Conclusion:**

In this report, we describe a typical clinical manifestation of an uncommon cause of acute myocardial infarction. The dissection was started by an extreme physical effort, which is a known triggering factor. Management of these cases is always challenging because there are no evidence-based therapies or guideline-based recomendations.

**Table:** 

Abbreviations, acronyms & symbols	
ACS	= Acute coronary syndrome
CABG	= Coronary artery bypass grafting
LAD	= Left anterior descending
PCI	= Percutaneous coronary intervention
SCAD	= Spontaneous coronary artery dissection

## PATIENT CHARACTERIZATION

A previously healthy 48-year-old male farmer was admitted to the emergency room complaining of anterior chest pain described as "tearing", which started after extreme physical exertion, co-occurring with general malaise and lipothymia.

He denied having previous comorbidities such as hypertension, diabetes, family history of cardiovascular disease and smoking or alcohol consumption.

Anterior wall ST-segment depression was observed in the electrocardiogram and troponin levels were increased.

**Fig. 1:**
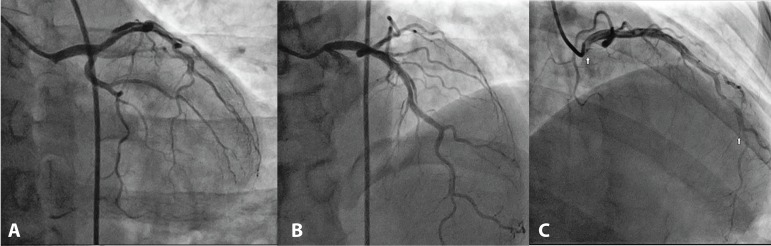
Spontaneous coronary artery dissection affecting the left anterior descending coronary artery. (A) Characteristic angiographic flap is visualized. (B) Long lesion is visualized. (C) Arrows pointing the initial and final dissection points.

The patient then underwent coronary catheterization. Angiography showed a tortuous left anterior descending (LAD) coronary artery with a dissection line involving proximal and middle segments, resulting in mild to moderate luminal stenosis; these findings were consistent with spontaneous coronary artery dissection (SCAD) diagnosis (Figure 1).

SCAD is a spontaneous separation between the layers of a coronary artery wall, non-iatrogenic or trauma related^[1]^, which has some predisposing factors, namely: fibromuscular dysplasia, postpartum status, multiparity, connective tissue disorders, systemic inflammatory conditions and hormonal therapy^[2-4]^.

SCAD is responsible for 0.1% to 0.4% of all acute coronary syndrome (ACS) cases in general population^[5,6]^, and up to a quarter of them in women ≤50 years old^[7]^.

Emotional and physical stressors were identified as common triggers in the Vancouver General Hospital SCAD registry. Out of 204 cases, 99 (48.5%) of them reported emotional stressors and 87 (42.6%) of them physical stressors prior to the SCAD event^[8]^.

In this report, we describe a typical case of effort-induced LAD coronary artery dissection in a middle-aged man, former smoker and with no other risk factors.

## DESCRIPTION OF THE TECHNIQUE EMPLOYED

At first, a conservative approach was chosen: administration of anticoagulation for 2 weeks with low-weight heparin (enoxaparin 1 mg/kg, subcutaneously, twice a day) and then statin, ticagrelor and beta-blocker.

**Fig. 2:**
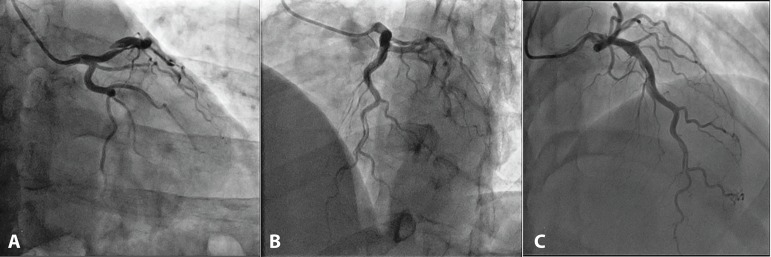
Control angiogram three months after the initial episode. (A) Increased dissection flap observed. (B) Dissection flap extension. (C) Involvement of diagonal branches.

Control cardiac catheterization, 3 months later, showed dissection progression to the distal segment (Figure 2, Movies 1 and 2). Considering the dissection extension and technical difficulty for percutaneous coronary intervention (PCI), he was referred to surgical intervention.

**Movies 1 and 2:**
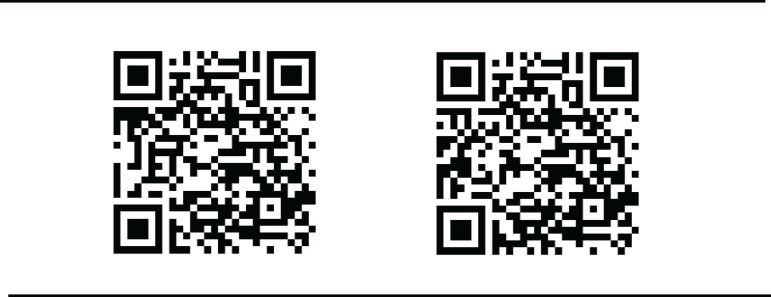
Spontaneous coronary artery dissection affecting the left anterior descending coronary artery.

Coronary artery bypass grafting (CABG) was performed under mild hypothermia, extracorporeal circulation, and aortic clamping. The internal thoracic artery was anastomosed in the distal portion of the LAD coronary artery, a dissection-free segment, and the great saphenous vein was anastomosed in a large-caliber 2^nd^ diagonal branch, whose origin was in the false lumen. Cardiopulmonary bypass and cross-clamping times were 35 and 26 minutes, respectively.

Although conservative management has generally been associated with favorable outcomes, it is also associated with small hazard of dissection progression and the consequent need for intervention^[9]^. Thus, the decision for revascularization, with PCI or CABG, should rely on clinical status, hemodynamic instability, and angiographic characteristics.

Comparing conservative *versus* aggressive management, Shamloo et al.^[10]^ described that 21.2% of conservatively managed patients eventually required surgical or catheter-based interventions, but only 2.5% of the patients initially treated with an invasive strategy needed that. Those patients with an isolated single lesion in left or right coronary artery had a statistically significant better outcome when treated with early aggressive strategy.

For our patient, we had to adopt an invasive strategy due to the dissection distal progression after three months of optimized clinical treatment. Successful surgical revascularization was achieved and the patient had an uneventful postoperative course. He was discharged on the seventh postoperative day, taking aspirin (100 mg, daily), beta-blocker (metoprolol succinate 50 mg, daily) and statin (rosuvastatin 10 mg, daily).

## CONCLUSION

We reported a typical clinical manifestation of a rare cause of acute myocardial infarction, especially in men. In this case, SCAD was clearly initiated by an extreme physical exertion, which has been described as an important triggering factor. After an initial conservative approach, the dissection progressed to the distal segment, and we opted for surgical treatment, which was successful. Management of these cases is always challenging because there is no evidence-based therapies or recommendations based on guidelines.

**Table:** 

Authors' roles & responsibilities	
APT	Conception and study design; realization of the operation; manuscript redaction or critical review of its content; final manuscript approval
ANK	Conception and study design; realization of the operation; manuscript redaction or critical review of its content; final manuscript approval
LEPR	Conception and study design; realization of the operation; manuscript redaction or critical review of its content; final manuscript approval
OCBW	Conception and study design; realization of the operation; manuscript redaction or critical review of its content; final manuscript approval
